# Machine learning identifies PPARG as a diagnostic biomarker for sepsis linked to CD14/NF-κB signaling: integrated transcriptomics and experimental validation

**DOI:** 10.3389/fcimb.2026.1800050

**Published:** 2026-05-28

**Authors:** Yingying Ji, Xin Xiao, Yanou Li, Hua Meng, Fang Huang, Jun Wang

**Affiliations:** Department of Critical Care Medicine, The First Affiliated Hospital of Soochow University, Suzhou, China

**Keywords:** sepsis, diagnostic biomarker, PPARG, CD14, NF-κB, machine learning, immunometabolism

## Abstract

**Background:**

Sepsis is a life-threatening organ dysfunction caused by a dysregulated host response to infection. Early diagnosis remains challenging due to substantial clinical and biological heterogeneity. CD14 is a central pattern-recognition receptor in innate immune activation, but the downstream network linking CD14 to immunometabolic regulation remains incompletely defined. We aimed to identify CD14-associated blood-based diagnostic biomarkers for sepsis and explore potential regulatory mechanisms.

**Methods:**

Whole-blood transcriptomic datasets were retrieved from the Gene Expression Omnibus. GSE236713 was the discovery cohort and GSE65682 the external validation cohort. Candidate genes were identified through overlap of differentially expressed genes between *CD14*-high and *CD14*-low samples and sepsis-related WGCNA modules. Hub genes were prioritized by protein-protein interaction analysis, and feature genes were identified by LASSO and random forest. Multiple machine learning algorithms were compared, and an artificial neural network (ANN) classifier was established. Clinical associations, immune cell composition (CIBERSORT), and pathway activity (GSEA) were assessed. Mechanistic validation was performed in LPS-stimulated RAW264.7 macrophages using CD14 knockout/overexpression and SN50-mediated inhibition of NF-κB nuclear translocation.

**Results:**

Five feature genes (*MMP9*, *PPARG*, *C1QC*, *MS4A4A*, and *ARG1*) were identified. Among seven algorithms, the ANN achieved the best performance (internal AUC = 0.974, external AUC = 0.953). *PPARG* showed the strongest single-gene diagnostic ability (AUC = 0.994) and correlated with SOFA score (*r* = 0.50, *P* = 1.5 × 10^-^²^9^), and was prioritized as a diagnostic biomarker. In a Cox model, *PPARG* was associated with ICU short-term outcome risk (HR = 1.64, 95% CI 1.34–2.01, *P* = 1.3 × 10^-6^). GSEA indicated enrichment of NF-κB-related pathways in samples with low *PPARG* expression. CIBERSORT suggested the strongest correlation between *PPARG* and monocyte proportion (*r* = 0.328, *P* = 1.06 × 10^-6^). *In vitro*, LPS-induced PPARG downregulation was CD14-dependent, and SN50 partially reversed PPARG suppression and reduced TNF-α secretion in CD14-overexpressing cells.

**Conclusion:**

By integrating machine learning with experimental validation, this study prioritized PPARG as a diagnostic biomarker for sepsis and provided supportive evidence for its association with CD14/NF-κB signaling. These findings offer a basis for developing host-response-based diagnostic signatures and further investigation of PPARG-related immunometabolic regulation in sepsis.

## 1 Introduction

Sepsis is a life-threatening organ dysfunction caused by a dysregulated host response to infection and remains a major global health challenge owing to its high incidence and mortality (Singer et al., 2016; Rudd et al., 2020; Brixey et al., 2024). Early recognition is difficult due to heterogeneous and often nonspecific clinical manifestations, and delays in diagnosis and treatment are common in clinical practice (Schinkel et al., 2023). Widely used inflammatory biomarkers, including C-reactive protein (CRP) and procalcitonin (PCT), show suboptimal sensitivity and specificity in the early phase of sepsis (Huang et al., 2023). Therefore, there is an unmet need for reproducible diagnostic biomarkers with improved diagnostic performance, along with a better mechanistic understanding of how such biomarkers relate to key immune pathways (Giamarellos-Bourboulis et al., 2024), to enable earlier intervention and more precise patient stratification.

CD14 is a critical pattern-recognition receptor that recognizes pathogen-associated molecular patterns such as lipopolysaccharide (LPS) and cooperates with Toll-like receptor 4 (TLR4) to initiate innate immune signaling (Chen et al., 2018; Sharygin et al., 2023). Excessive activation of innate immunity is a hallmark of early sepsis pathophysiology, in which the CD14-TLR4-NF-κB axis contributes to amplification of inflammatory cascades (Kabanov et al., 2020; Ciesielska et al., 2021). However, prior studies have largely emphasized the inflammation-initiating role of CD14, while the downstream CD14-associated transcriptional network remains insufficiently characterized. In particular, it remains unclear which CD14-related molecules may represent robust early diagnostic biomarker candidates and whether CD14-driven signaling modulates key effector genes, especially those involved in immunometabolic regulation and related regulatory pathways, including those linked to PPARG (Liu et al., 2023; Bruno et al., 2025). This knowledge gap limits the development of CD14-network-based diagnostic signatures, risk stratification tools, and potential therapeutic targets.

**Figure 1:**
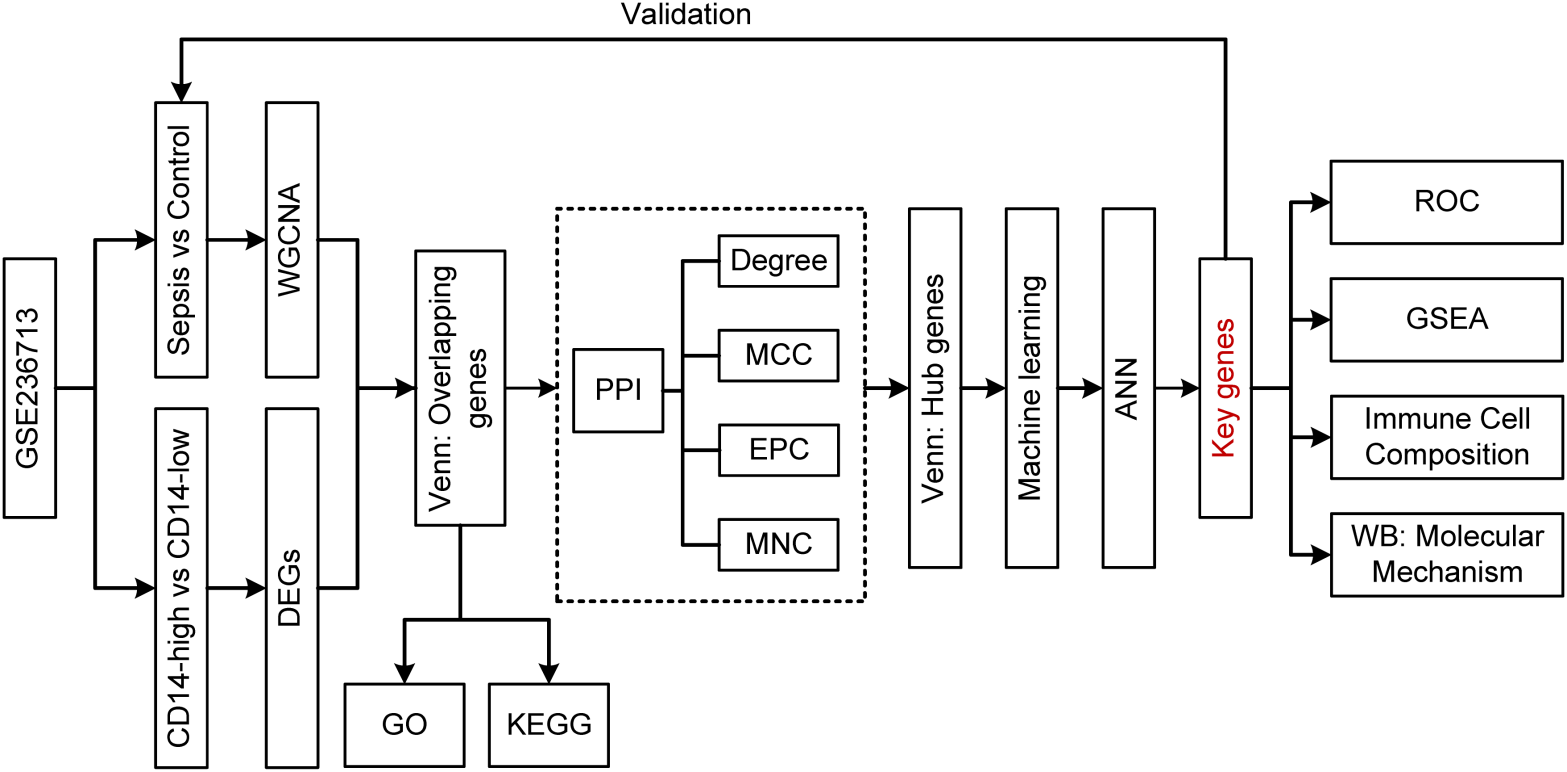
Study workflow for feature gene identification and prioritization of PPARG as a diagnostic biomarker linked to the CD14/NF-κB axis in sepsis.

To address these issues, we employed an integrated workflow combining transcriptomic analysis, machine learning, and targeted experimental validation to systematically identify CD14-associated diagnostic biomarker candidates for sepsis. Public whole-blood transcriptomic cohorts were analyzed, and all samples were collected within 5 days of ICU admission to capture early-stage features. By integrating CD14 expression-related transcriptomic patterns with sepsis-associated molecular signals, we aimed to prioritize biomarkers with both diagnostic relevance and biological plausibility, followed by external validation in an independent cohort (Stanski and Wong, 2020; Yan et al., 2022). Immune cell composition and pathway-level changes were further evaluated to provide biological context for the identified candidates, and the overall study design is summarized in **Figure 1**. Given the growing recognition of crosstalk between inflammation and metabolic reprogramming in sepsis (Venet and Monneret, 2018; Willmann and Moita, 2024), we further focused on PPARG within the CD14-associated network because of its potential relevance to immunoregulatory and metabolic processes (Chen et al., 2025), aiming to provide a basis for early molecular diagnosis and mechanistic investigation.

## 2 Materials and methods

### 2.1 Data sources

All transcriptomic datasets were obtained from the Gene Expression Omnibus (GEO) database (https://www.ncbi.nlm.nih.gov/geo/). The discovery cohort GSE236713 (platform: GPL17077) comprises whole-blood microarray profiles from 143 patients with sepsis (324 samples collected at ICU days 1, 2, and 5) and 30 healthy controls (Szakmany et al., 2023). The external validation cohort GSE65682 (platform: GPL13667) comprises whole-blood transcriptomic data from 760 patients with sepsis (sampled within 24 hours of ICU admission) and 42 healthy controls (Scicluna et al., 2017). All participants in both datasets were of European ancestry. Baseline clinical characteristics available from the public annotations, including age, sex, infection source, SOFA score, selected comorbidity information, and available outcome data, are summarized in **Supplementary Table 3**.

### 2.2 Identification of differentially expressed genes

All data processing and statistical analyses were performed in R (version 4.4.3). When multiple microarray probes mapped to the same gene symbol, probe-level expression values were collapsed to a single gene-level value by averaging. To assess the discriminative ability of CD14 for sepsis versus healthy controls, receiver operating characteristic (ROC) curves were generated using the pROC package. The optimal *CD14* expression cutoff was determined by maximizing the Youden index (best.method = “youden”) (Matysiak et al., 2025), yielding an AUC of 0.859, and was applied to dichotomize all samples in the GSE236713 cohort, including both sepsis patients and healthy controls, into *CD14*-high and *CD14*-low groups for identification of transcriptomic differences associated with *CD14* expression status. Differentially expressed genes (DEGs) between the two groups were identified using the limma package. Genes with |log_2_FC| > 0.5 and an adjusted *P* < 0.05 (Benjamini-Hochberg correction) were considered DEGs.

### 2.3 WGCNA analysis

A weighted gene co-expression network was constructed using the WGCNA package in R. To reduce noise and improve computational efficiency, the top 25% most variable genes were retained for downstream analysis (Mucalo Katunaric et al., 2025). The soft-thresholding power (β) was selected using the pickSoftThreshold function, and the chosen β achieved a scale-free topology model fit index (R²) of 0.85. An unsigned adjacency matrix was constructed and transformed into a topological overlap matrix (TOM). Hierarchical clustering with average linkage was performed based on TOM dissimilarity (1 − TOM). Gene modules were identified using the dynamic tree cut algorithm with a minimum module size of 30 genes. Closely related modules were merged using a cut height of 0.2, equivalent to an eigengene correlation threshold of 0.8. Module-trait relationships were assessed by correlating module eigengenes with clinical traits. For modules significantly associated with the trait of interest, gene significance (GS) was defined as the correlation between each gene and the phenotype, and module membership (MM) was defined as the correlation between each gene and the corresponding module eigengene.

### 2.4 Functional enrichment analysis

To identify candidate genes associated with both *CD14* expression status and the sepsis phenotype, the overlap between DEGs and genes from sepsis-associated WGCNA modules was identified, and the overlapping set was defined as candidate genes. Gene Ontology (GO) and Kyoto Encyclopedia of Genes and Genomes (KEGG) pathway enrichment analyses were performed for these candidate genes using Metascape (http://metascape.org), with *P* < 0.05 and an enrichment factor > 1.5 as significance thresholds (Zhou et al., 2019; Zhang et al., 2022).

Gene set enrichment analysis (GSEA) was performed using the clusterProfiler package to explore pathways associated with the prioritized diagnostic biomarker and to examine whether these associations differed by *CD14* expression status. Reactome gene sets from the Molecular Signatures Database (MSigDB) were used as the reference collection. GSEA was conducted in two steps. First, all 354 samples were divided into high- and low-expression groups according to the mean expression of the diagnostic biomarker. Second, samples were stratified into *CD14*-high and *CD14*-low groups as defined in Section 2.2, and within each stratum, samples were further dichotomized into high- and low-expression subgroups based on diagnostic biomarker expression. Normalized enrichment scores (NES) were calculated to compare the direction and magnitude of pathway enrichment across groups.

### 2.5 PPI network construction and topological analysis

A protein-protein interaction (PPI) network was constructed for the candidate genes using the STRING database (https://string-db.org/), with the minimum required interaction score set to 0.7 (high confidence). The network was visualized in Cytoscape (version 3.10.3).

To identify robust hub genes, network topology was assessed using the cytoHubba plugin with four algorithms: maximal clique centrality (MCC), maximum neighborhood component (MNC), edge percolated component (EPC), and degree. The top 50 genes ranked by each algorithm were retained, and genes present across all four lists were defined as hub genes for subsequent machine learning-based feature gene selection.

### 2.6 Machine learning-based feature gene selection and clinical relevance assessment

To identify robust feature genes with high predictive value, hub genes identified in Section 2.5 were subjected to feature selection using multiple machine learning approaches. Least absolute shrinkage and selection operator (LASSO) regression was performed using the glmnet package. The penalty parameter (λ) was selected by 10-fold cross-validation, and genes were retained at λmin, the value yielding the minimum cross-validation error. In parallel, a random forest (RF) model was fitted using the randomForest package (ntree = 500). Feature importance was ranked by the mean decrease in Gini index, and genes with a score > 2.0 were retained. Genes selected by both LASSO and RF were defined as feature genes. These feature genes were then used as the input variables for subsequent classifier comparison and ANN model development.

For diagnostic model development, the discovery cohort (GSE236713) was randomly split into a training set and an internal validation set at a 7:3 ratio using the caret package. Seven classifiers were compared: artificial neural network (ANN), extreme gradient boosting (XGB), gradient boosting machine (GBM), support vector machine (SVM), k-nearest neighbors (KNN), decision tree (DT), and generalized linear model (GLM). To ensure comparability, all models were trained under the same caret resampling framework using 10-fold cross-validation (repeated once) on the training set, and performance was evaluated primarily by the area under the ROC curve (AUC). Model tuning followed the default caret settings for each algorithm. After identifying the best-performing algorithm, a final ANN model was implemented using the neuralnet package and retrained on all samples in GSE236713 (*n* = 354). The final network contained two hidden layers with 4 and 2 neurons, respectively, and was trained using resilient backpropagation with backtracking (rprop+). Training was terminated when either the sum of absolute partial derivatives of the error with respect to the weights fell below the default threshold of 0.01 or the maximum number of training steps (stepmax = 100,000) was reached, whichever occurred first. The model converged at 92,494 steps with a final training error of 0.003255. Generalizability was subsequently assessed in the external validation cohort (GSE65682).

To evaluate clinical relevance, Spearman correlation analysis was used to assess associations between feature gene expression and Sequential Organ Failure Assessment (SOFA) scores. In datasets with available outcome data, multivariable Cox regression was performed to evaluate associations between feature genes and short-term prognosis.

### 2.7 Immune cell landscape analysis and its correlation with PPARG

To characterize the immune cell landscape in early sepsis, the relative proportions of 22 immune cell subsets in GSE236713 were estimated using the CIBERSORT algorithm with 1,000 permutations. Spearman correlation was used to evaluate associations between *PPARG* expression and the estimated proportions of immune cell subsets. To determine whether the association between *PPARG* expression and monocyte fraction was independent of disease status, a multivariable linear regression model including sepsis status as a covariate was additionally performed. The correlation between *PPARG* expression and monocyte fraction was also examined within sepsis samples alone. Figures were generated using GraphPad Prism (version 9.5.0). Box plots were used to compare immune cell proportions between groups, lollipop plots to display the strength and significance of correlations, and scatter plots to visualize PPARG-monocyte associations.

### 2.8 Cell culture and lentiviral transduction

The murine macrophage cell line RAW264.7 was obtained from the American Type Culture Collection (ATCC). Cells were cultured in DMEM (KeyGEN BioTECH, China) supplemented with 10% fetal bovine serum (FBS; Gibco, USA), 100 U/mL penicillin, and 100 µg/mL streptomycin, and maintained at 37 °C in a humidified incubator with 5% CO_2_. To establish an *in vitro* sepsis-like inflammatory model, RAW264.7 cells were stimulated with lipopolysaccharide (LPS; 100 ng/mL) for 6 h (Zeng et al., 2022). For NF-κB inhibition, cells were pretreated with the NF-κB/Rel nuclear translocation inhibitor SN50 (10 µM; Sigma-Aldrich, USA) for 4 h prior to LPS stimulation (Wu et al., 2020).

To manipulate CD14 expression, RAW264.7 cells were transduced with lentiviral vectors. CD14 knockout was achieved using a lentiviral CRISPR/Cas9 construct carrying a CD14-targeting sgRNA (5′-GAACCTCCGCAACGTGTCGT-3′), together with a matched negative control vector (GeneChem, China). For CD14 overexpression, a lentiviral vector encoding the full-length murine CD14 transcript (NM_009841.4; catalog no. CV186) and the matched empty vector were purchased from GeneChem. Cells were transduced for 48 h and subsequently selected with puromycin (3 µg/mL) for 3 days to eliminate non-transduced cells. The efficiency of CD14 knockout and overexpression in the resulting stable cell pools was confirmed by western blotting prior to subsequent experiments.

### 2.9 Western blot

Total cellular protein was extracted using Western and IP lysis buffer (cat. no. P0013; Beyotime, China) supplemented with a protease inhibitor cocktail. Protein lysates were separated by SDS-PAGE and transferred to polyvinylidene difluoride (PVDF) membranes (Millipore, USA). Membranes were blocked with 5% non-fat milk at room temperature for 1 h to reduce nonspecific binding, then incubated with primary antibodies at 4 °C overnight. Details of all primary antibodies are provided in **Supplementary Table 1**. Membranes were subsequently incubated with an HRP-conjugated goat anti-rabbit secondary antibody (cat. no. A0208; 1:1,000; Beyotime, China) at room temperature for 1 h. After three washes, protein bands were detected using ECL Prime reagent and imaged according to the manufacturer’s instructions.

### 2.10 ELISA

Tumor necrosis factor-α (TNF-α) levels in cell culture supernatants were quantified using a TNF-α ELISA kit (cat. no. PT512; Beyotime, China) according to the manufacturer’s instructions. Supernatants from RAW264.7 cells in each experimental group were collected, and absorbance at 450 nm was measured using a Bio-Rad microplate reader. TNF-α concentrations were calculated from a standard curve. All measurements were performed in triplicate.

### 2.11 Statistical analysis

Bioinformatics analyses were performed in R (version 4.4.3), and experimental data were analyzed using GraphPad Prism (version 9.5.0). Quantitative data are presented as mean ± standard deviation (SD) from at least three independent experiments. For comparisons between two groups, Student’s t-test was used for normally distributed data, and the Wilcoxon rank-sum test for non-normally distributed data. For comparisons among multiple groups, one-way analysis of variance (ANOVA) was applied. A *P* value < 0.05 was considered statistically significant.

## 3 Results

### 3.1 Identification of candidate genes by differential expression analysis and WGCNA

**Figure 2:**
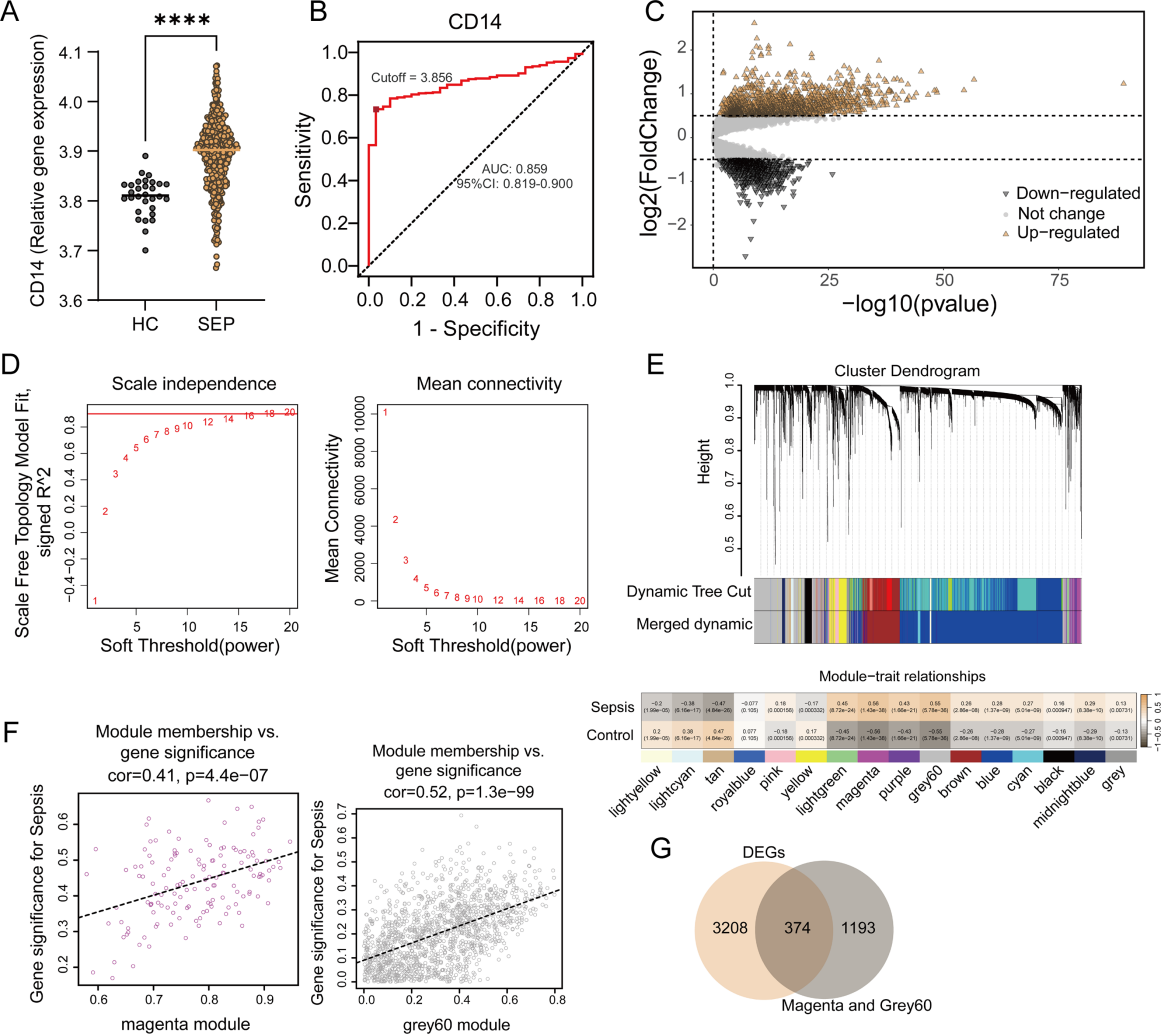
Identification of candidate genes by differential expression analysis and WGCNA in GSE236713. **(A)** *CD14* mRNA expression in sepsis versus healthy controls. **(B)** ROC curve evaluating CD14 for discriminating sepsis from controls; the optimal cutoff (3.856) used to define *CD14*-high and *CD14*-low groups is indicated. **(C)** Volcano plot of DEGs between *CD14*-high and *CD14*-low groups. **(D)** Selection of soft-thresholding power (β) for WGCNA based on scale-free topology fit and mean connectivity; *β* = 16 was selected. **(E)** WGCNA dendrogram of clustered genes with module assignments and module-trait relationship heatmap. **(F)** Relationships between gene significance (GS) and module membership (MM) in the magenta and grey60 modules. GS reflects the correlation of each gene with the sepsis phenotype, and MM reflects the correlation of each gene with the corresponding module eigengene. **(G)** Venn diagram showing the overlap between DEGs and genes from the sepsis-associated WGCNA modules (magenta and grey60), yielding 374 candidate genes associated with both *CD14* expression status and the sepsis phenotype.

After normalization of the GSE236713 expression matrix, *CD14* expression was compared between patients with sepsis and healthy controls. *CD14* expression was significantly higher in sepsis (**Figure 2A**). To assess whether early sampling-time heterogeneity after ICU admission might affect *CD14* expression, a time-stratified analysis was performed across the D1, D2, and D5 sepsis subgroups. *CD14* expression was significantly elevated in all three subgroups compared with healthy controls, whereas no significant difference was observed among D1, D2, and D5, suggesting that *CD14* upregulation remained relatively stable during the first 5 days after ICU admission (**Supplementary Figure 1**). ROC analysis indicated that CD14 discriminated sepsis from controls with an AUC of 0.859 (95% CI 0.819–0.900), and the optimal cutoff determined by the Youden index was 3.856 (**Figure 2B**). Using this cutoff, all samples in the GSE236713 cohort, including both sepsis patients and healthy controls, were dichotomized into *CD14*-high and *CD14*-low groups for differential expression analysis (|log_2_FC| > 0.5 and adjusted *P* < 0.05; **Figure 2C**). A total of 3,582 DEGs were identified, comprising 2,538 upregulated and 1,044 downregulated genes.

To identify co-expression modules associated with sepsis, a weighted gene co-expression network was constructed using WGCNA. A soft-thresholding power of 16 was selected to achieve a scale-free topology fit index of R² = 0.85 (**Figure 2D**), and 16 modules were identified by dynamic tree cutting (**Figure 2E**). Module-trait analysis showed that the magenta and grey60 modules were most positively correlated with sepsis status (magenta: *r* = 0.56, *P* = 1.43 × 10^-^³^8^; grey60: *r* = 0.55, *P* = 5.78 × 10^-^³^6^). The relationship between gene significance (GS) and module membership (MM) was further examined. GS was defined as the correlation between an individual gene and the sepsis phenotype, reflecting the strength of association with sepsis, whereas MM was defined as the correlation between an individual gene and the corresponding module eigengene, reflecting how well that gene represented the overall expression pattern of the module. The GS-MM plots were used to assess whether genes more central to a given module also tended to be more strongly associated with sepsis. In the magenta module (141 genes), GS was positively correlated with MM (*r* = 0.41, *P* = 4.4 × 10^-7^), and a similar pattern was observed in the grey60 module (1,426 genes; *r* = 0.52, *P* = 1.3 × 10^-99^) (**Figure 2F**), suggesting that intramodular hub genes in these modules were also more strongly associated with the sepsis phenotype.

DEGs were then intersected with genes from the sepsis-associated modules (magenta and grey60), yielding 374 candidate genes associated with both *CD14* expression status and the sepsis phenotype (**Figure 2G**), which were carried forward for subsequent analyses. Gene lists at each filtering step are provided in **Supplementary Table 2**.

### 3.2 Functional enrichment and PPI-based identification of hub genes

**Figure 3:**
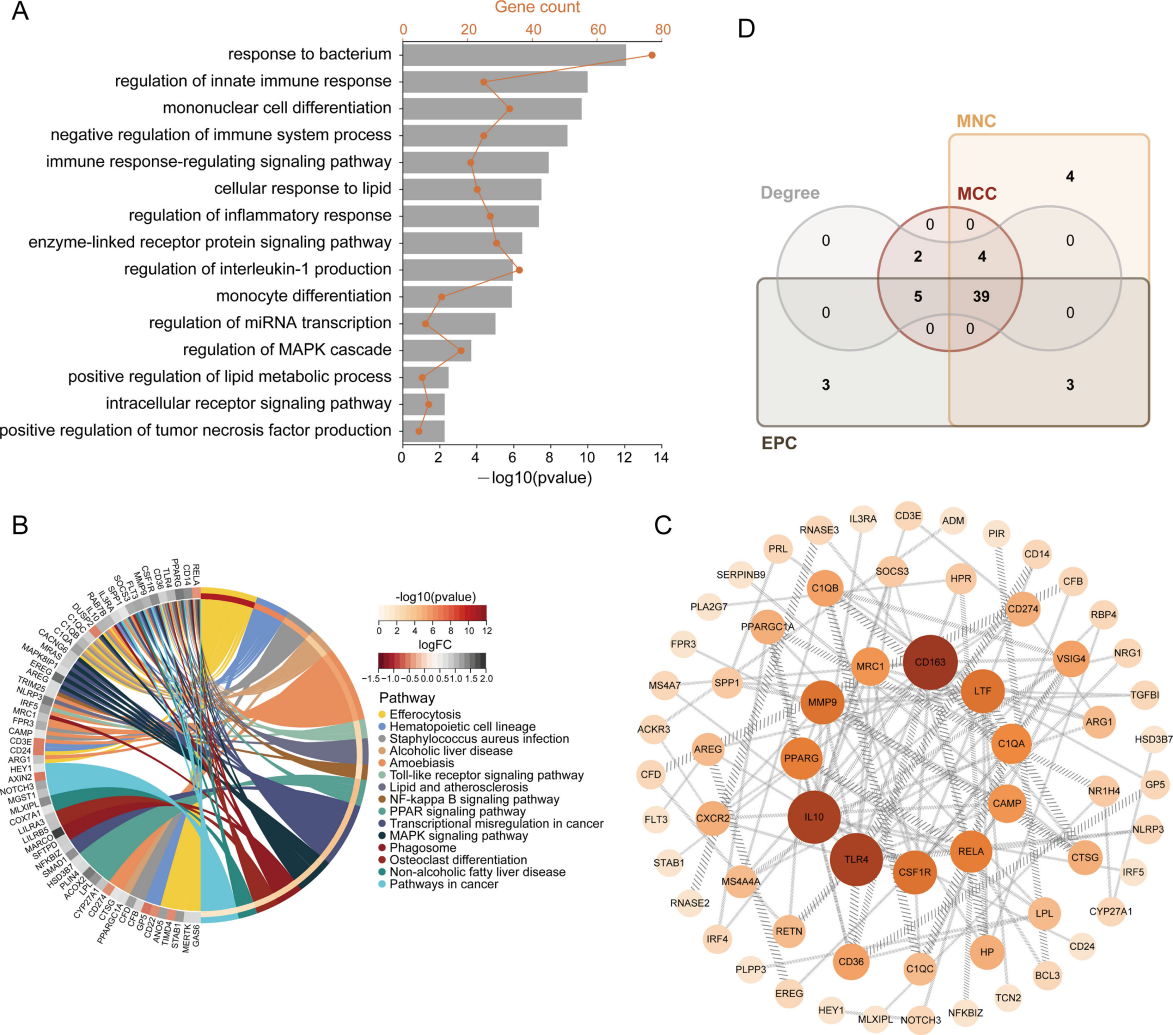
Functional enrichment analysis and PPI network-based prioritization of hub genes from candidate genes associated with both *CD14* expression status and the sepsis phenotype. **(A)** GO enrichment analysis of the 374 candidate genes, showing the top enriched biological process terms. **(B)** KEGG pathway enrichment results for the 374 candidate genes, visualized as a pathway-gene interaction chord diagram. **(C)** PPI network of the candidate genes constructed using STRING (minimum required interaction score = 0.7) and visualized in Cytoscape to assess protein-level functional interactions and prioritize highly interconnected genes. Of the 374 candidate genes, 270 were retained in the network. Node color intensity reflects degree (darker red indicates higher degree), and edge width represents interaction confidence (thicker edges indicate stronger interactions). **(D)** Venn diagram showing the intersection of the top 50 genes ranked by four cytoHubba algorithms (Degree, MCC, MNC, and EPC), yielding 39 hub genes for downstream machine learning-based feature gene selection.

To explore the biological functions of the 374 candidate genes, GO and KEGG enrichment analyses were performed. GO biological process terms were primarily enriched in immune- and inflammation-related processes, including pathogen response, regulation of inflammatory responses, and innate immune regulation, consistent with the pattern of immune dysregulation in sepsis. Lipid-related responses and lipid metabolic processes were also enriched, suggesting a potential link between inflammatory activation and metabolic reprogramming (**Figure 3A**). KEGG analysis indicated enrichment of canonical inflammatory pathways, including NF-κB signaling, Toll-like receptor signaling, and MAPK signaling, as well as pathways related to lipid metabolism and nuclear receptor signaling, implying possible crosstalk between inflammation and lipid metabolic regulation (**Figure 3B**).

To further prioritize hub genes from the 374 candidate genes, these genes were evaluated from the perspective of protein-level functional interactions. Although the DEG and WGCNA overlap analysis identified candidate genes associated with both *CD14* expression status and the sepsis phenotype, this step was based primarily on transcript-level differential expression and co-expression patterns and did not capture known functional connectivity among the encoded proteins. A PPI network was therefore constructed as an additional prioritization step to identify highly interconnected genes occupying more central positions in the relevant biological network, thereby improving the biological interpretability of downstream gene prioritization. The network was constructed using STRING with a high-confidence interaction threshold (minimum required interaction score = 0.7). Because some candidate genes could not be mapped to STRING or did not form interactions meeting the predefined threshold, the resulting network contained 270 of the 374 candidate genes and 146 edges, with an average node degree of 1.08 (**Figure 3C**). The remaining 104 candidate genes were not included in the final PPI network. As STRING is a curated protein interaction resource, this step may partly bias the network toward genes with better-characterized protein interactions, including well-studied immune-related genes, which should be considered when interpreting the hub gene prioritization results. Hub genes were ranked using four cytoHubba algorithms, and the top 50 genes from each algorithm were intersected to reduce algorithm-specific bias and improve selection robustness. This procedure yielded 39 hub genes (**Figure 3D**, **Table 1**), which were carried forward for machine learning-based feature gene selection.

**Table 1:** Hub genes identified by PPI network analysis and their topological scores.

Gene symbol	Gene name	logFC	MCC	MNC	EPC	Degree
TGFBI	transforming growth factor, beta-induced, 68kDa	1.465	2	2	9.362	2
HP	haptoglobin	1.432	5	2	14.017	5
PPARG	peroxisome proliferator-activated receptor gamma	1.375	28	9	18.943	9
VSIG4	V-set and immunoglobulin domain containing 4	1.370	72	6	15.898	6
NLRP3	NLR family, pyrin domain containing 3	1.348	2	2	10.005	2
HPR	haptoglobin-related protein	1.269	3	2	8.901	3
CSF1R	colony stimulating factor 1 receptor	1.242	65	9	18.499	10
CD14	CD14 molecule	1.231	2	2	10.211	2
RETN	resistin	1.221	4	2	11.111	4
LPL	lipoprotein lipase	1.212	7	3	11.245	4
CD163	CD163 molecule	1.162	87	13	20.667	16
C1QA	complement component 1, q subcomponent, A chain	1.128	79	7	16.980	8
MS4A4A	membrane-spanning 4-domains, subfamily A, member 4A	1.062	24	4	12.657	4
SOCS3	suppressor of cytokine signaling 3	1.035	6	3	13.079	3
FPR3	formyl peptide receptor 3	1.017	1	1	5.969	1
MMP9	matrix metallopeptidase 9	0.967	36	10	19.349	10
IL10	interleukin 10	0.962	54	11	20.585	15
C1QB	complement component 1, q subcomponent, B chain	0.940	72	6	16.058	6
TLR4	toll-like receptor 4	0.938	66	15	21.271	15
PRL	prolactin	0.938	2	2	7.832	2
MS4A7	membrane-spanning 4-domains, subfamily A, member 7	0.928	2	2	9.633	2
ARG1	arginase 1	0.852	12	4	14.682	4
MRC1	mannose receptor, C type 1	0.852	30	7	18.993	7
BCL3	B-cell lymphoma 3	0.784	2	1	6.009	2
CD36	CD36 molecule (thrombospondin receptor)	0.726	12	6	15.317	6
C1QC	complement component 1, q subcomponent, C chain	0.723	24	4	13.427	4
CXCR2	chemokine (C-X-C motif) receptor 2	0.688	8	3	14.352	5
CFB	complement factor B	0.675	2	1	5.954	2
AREG	amphiregulin	0.637	4	2	7.443	4
PPARGC1A	peroxisome proliferator-activated receptor gamma, coactivator 1 alpha	0.573	8	3	11.744	5
CAMP	cathelicidin antimicrobial peptide	0.541	21	6	17.177	7
CTSG	cathepsin G	0.528	8	3	12.293	5
ACKR3	atypical chemokine receptor 3	0.528	2	1	5.353	2
SPP1	secreted phosphoprotein 1	0.527	4	3	10.651	3
RELA	v-rel avian reticuloendotheliosis viral oncogene homolog A	-0.507	19	6	17.729	9
LTF	lactotransferrin	-0.523	27	9	17.628	10
CD274	CD274 molecule	-0.545	9	4	14.988	5
CD3E	CD3e molecule, epsilon (CD3-TCR complex)	-0.633	2	2	9.248	2
GP5	glycoprotein V (platelet)	-0.691	2	2	9.910	2

The 39 hub genes were defined as the intersection of the top 50 genes ranked by four cytoHubba algorithms (Degree, MCC, MNC, and EPC). logFC denotes the log_2_ fold change between the *CD14*-high and *CD14*-low groups.

### 3.3 Machine learning–based identification of feature genes and ANN construction

**Figure 4:**
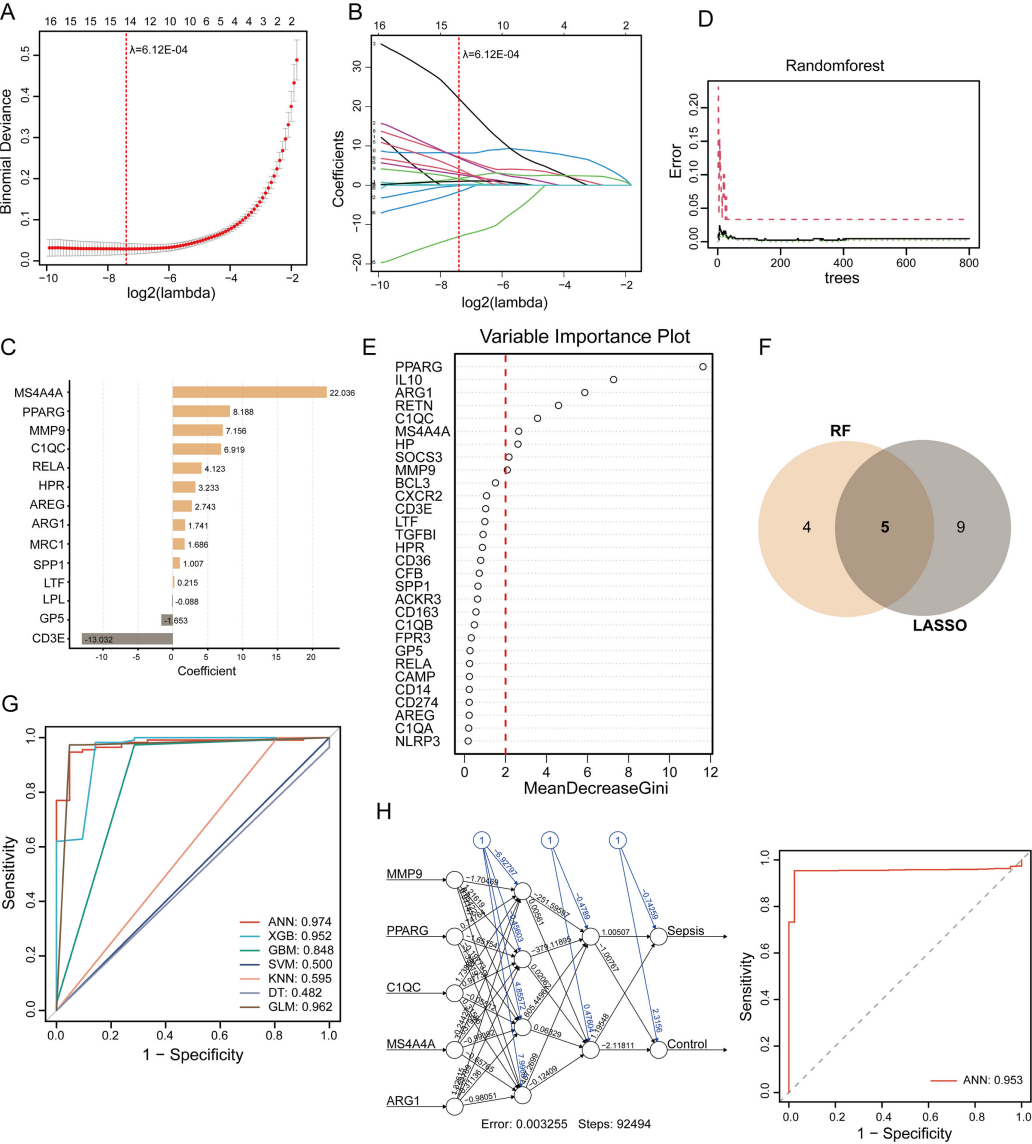
Machine learning-based identification of feature genes and construction of an ANN diagnostic model for sepsis. **(A)** Ten-fold cross-validation curve for LASSO regression showing the optimal penalty parameter (λ) used for feature selection. **(B)** LASSO coefficient profiles of the 39 hub genes across different λ values. **(C)** Coefficients of the 14 genes retained with non-zero coefficients at the selected λ. **(D)** Random forest model tuning showing the relationship between the number of trees and out-of-bag error; ntree = 500 was selected. **(E)** Random forest feature importance ranked by mean decrease in Gini index; genes with importance > 2.0 were retained. **(F)** Overlap between genes selected by LASSO and random forest, yielding five feature genes (*MMP9*, *PPARG*, *C1QC*, *MS4A4A*, and *ARG1*). **(G)** ROC curves comparing seven machine learning classifiers in the internal validation set derived from GSE236713. **(H)** Architecture of the final ANN model (five feature genes as input with two hidden layers) and ROC curve showing model performance in the external validation cohort (GSE65682; AUC = 0.953). The ANN was retrained on all samples in GSE236713 (*n* = 354) prior to evaluation in GSE65682.

To identify feature genes from the 39 hub genes, feature selection was performed using both LASSO regression and random forest (RF). LASSO retained 14 genes with non-zero coefficients at the optimal penalty parameter (λ = 6.12 × 10^-4^; **Figures 4A–C**). RF identified 9 genes based on the mean decrease in Gini index (**Figures 4D, E**). The intersection of the two gene sets yielded five feature genes: *MMP9*, *PPARG*, *C1QC*, *MS4A4A*, and *ARG1* (**Figure 4F**; **Table 2**). Using these five genes as input features, the classification performance of seven machine learning algorithms was compared. In the internal validation set (derived from the 7:3 split of GSE236713), the ANN achieved the best performance (AUC = 0.974), exceeding that of XGBoost (AUC = 0.952) (**Figure 4G**). An ANN model was therefore selected for diagnostic classification. The final ANN architecture comprised an input layer with five feature genes and two hidden layers with 4 and 2 neurons, respectively. The ANN was retrained on all samples in the discovery cohort (GSE236713; *n* = 354) and subsequently evaluated in the external validation cohort GSE65682, achieving an AUC of 0.953 (**Figure 4H**).

**Table 2:** Feature genes selected by LASSO and random forest.

Gene symbol	Gene name	logFC	RF	LASSO
PPARG	peroxisome proliferator-activated receptor gamma	1.375	11.631	8.188
MS4A4A	membrane-spanning 4-domains, subfamily A, member 4A	1.062	2.641	22.036
MMP9	matrix metallopeptidase 9	0.967	2.085	7.156
ARG1	arginase 1	0.852	5.867	1.741
C1QC	complement component 1, q subcomponent, C chain	0.723	3.561	6.919

The five feature genes were defined as the intersection of genes retained by LASSO regression and those selected by random forest. logFC denotes the log_2_ fold change between the *CD14*-high and *CD14*-low groups. RF importance was quantified by the mean decrease in Gini index.

### 3.4 Clinical association analysis prioritizes PPARG as a diagnostic biomarker

**Figure 5:**
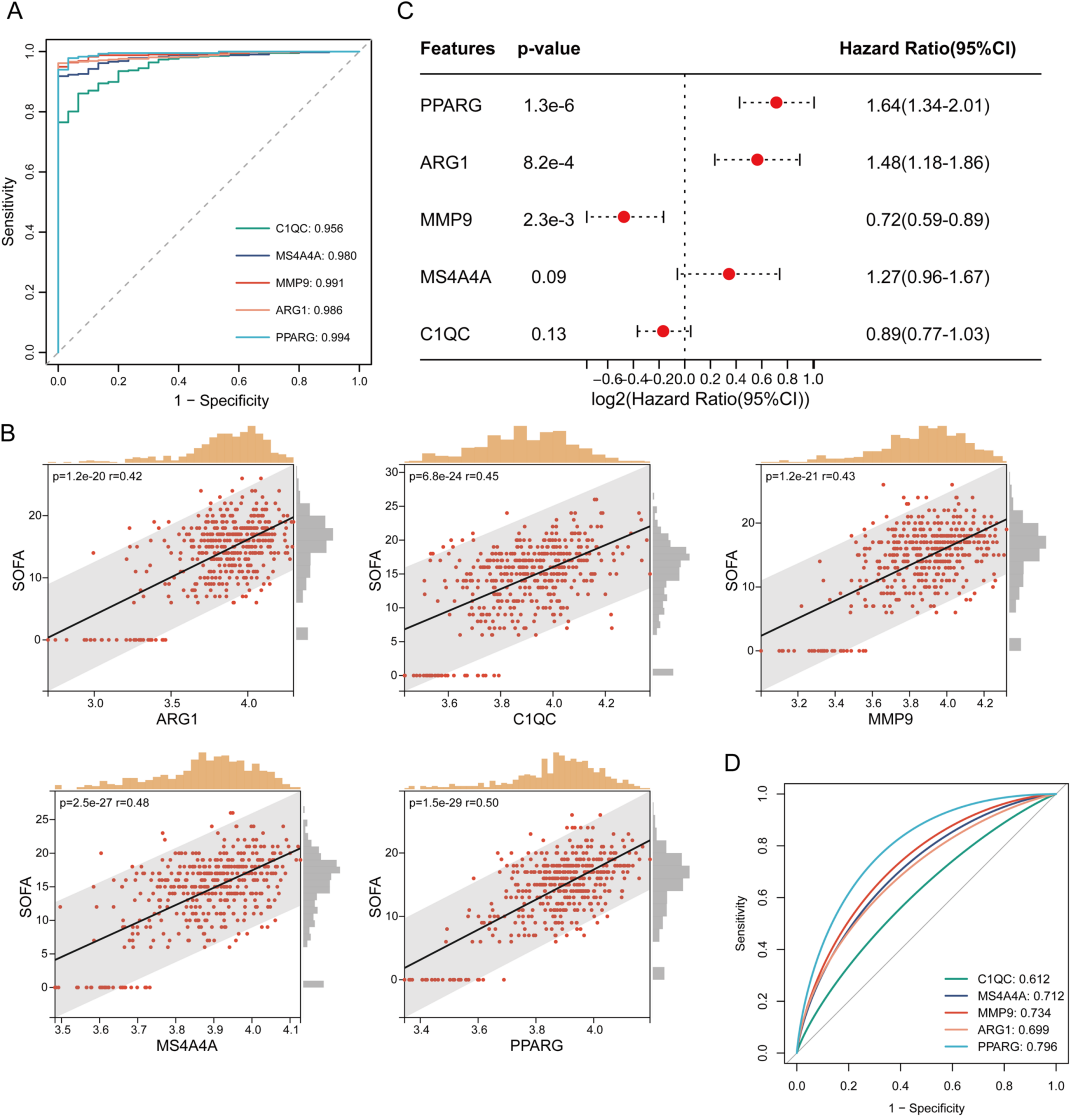
Clinical association analyses prioritize PPARG as a diagnostic biomarker. **(A)** ROC curves for the five feature genes (*MMP9*, *PPARG*, *C1QC*, *MS4A4A*, and *ARG1*) for discriminating sepsis from healthy controls in GSE236713. **(B)** Spearman correlations between expression of each feature gene and SOFA score in GSE236713. **(C)** Forest plot of the multivariable Cox regression model evaluating associations between the five feature genes and ICU short-term prognosis in GSE236713, shown as hazard ratios (HRs) with 95% confidence intervals. **(D)** ROC curves for the five feature genes for discriminating SOFA-defined risk groups within the sepsis cohort of GSE236713. Sepsis samples were stratified into a low/medium-risk group (SOFA < 11) and a high-risk group (SOFA ≥ 11). *PPARG* showed the highest discriminative performance among the five feature genes (AUC = 0.796).

To prioritize a clinically relevant target among the five feature genes, the single-gene diagnostic performance of each gene was assessed in the full GSE236713 cohort (*n* = 354). ROC analysis showed that PPARG had the highest discriminative ability and was therefore prioritized as a diagnostic biomarker for sepsis, while the remaining four genes also demonstrated high diagnostic performance (**Figure 5A**). Given that sepsis samples in GSE236713 were collected at different early time points (D1, D2, and D5), a time-stratified analysis of *PPARG* expression was performed. *PPARG* expression remained significantly different from that in healthy controls across all three subgroups. Pairwise comparisons showed no significant difference between D1 and D2 or between D2 and D5, whereas a modest difference was observed between D1 and D5, suggesting that *PPARG* remained dysregulated during the first 5 days after ICU admission, although some short-term temporal variation may exist (**Supplementary Figure 2**). *PPARG* expression was positively correlated with SOFA score (Spearman *r* = 0.50, *P* = 1.5 × 10^-^²^9^) (**Figure 5B**). To assess the association of feature genes with severity stratification within the sepsis cohort, supplementary ROC analyses were performed using a SOFA-based risk grouping strategy (< 11 vs ≥ 11). All five feature genes showed discriminative ability for distinguishing the higher-risk group, with *PPARG* achieving the highest AUC (AUC = 0.796) (**Figure 5D**). In a multivariable Cox regression model for ICU short-term outcome in the discovery cohort, *PPARG* was significantly associated with outcome risk (HR = 1.64, 95% CI 1.34–2.01, *P* = 1.3 × 10^-6^) (**Figure 5C**). The association between *PPARG* and 28-day survival in GSE65682 was also examined; although a similar overall trend was observed, it did not reach statistical significance (**Supplementary Figure 3**). Taken together, these findings support the diagnostic relevance of PPARG, while its associations with disease severity and outcome should be interpreted with caution and require validation in independent cohorts.

### 3.5 Immune cell composition and its association with PPARG expression

**Figure 6:**
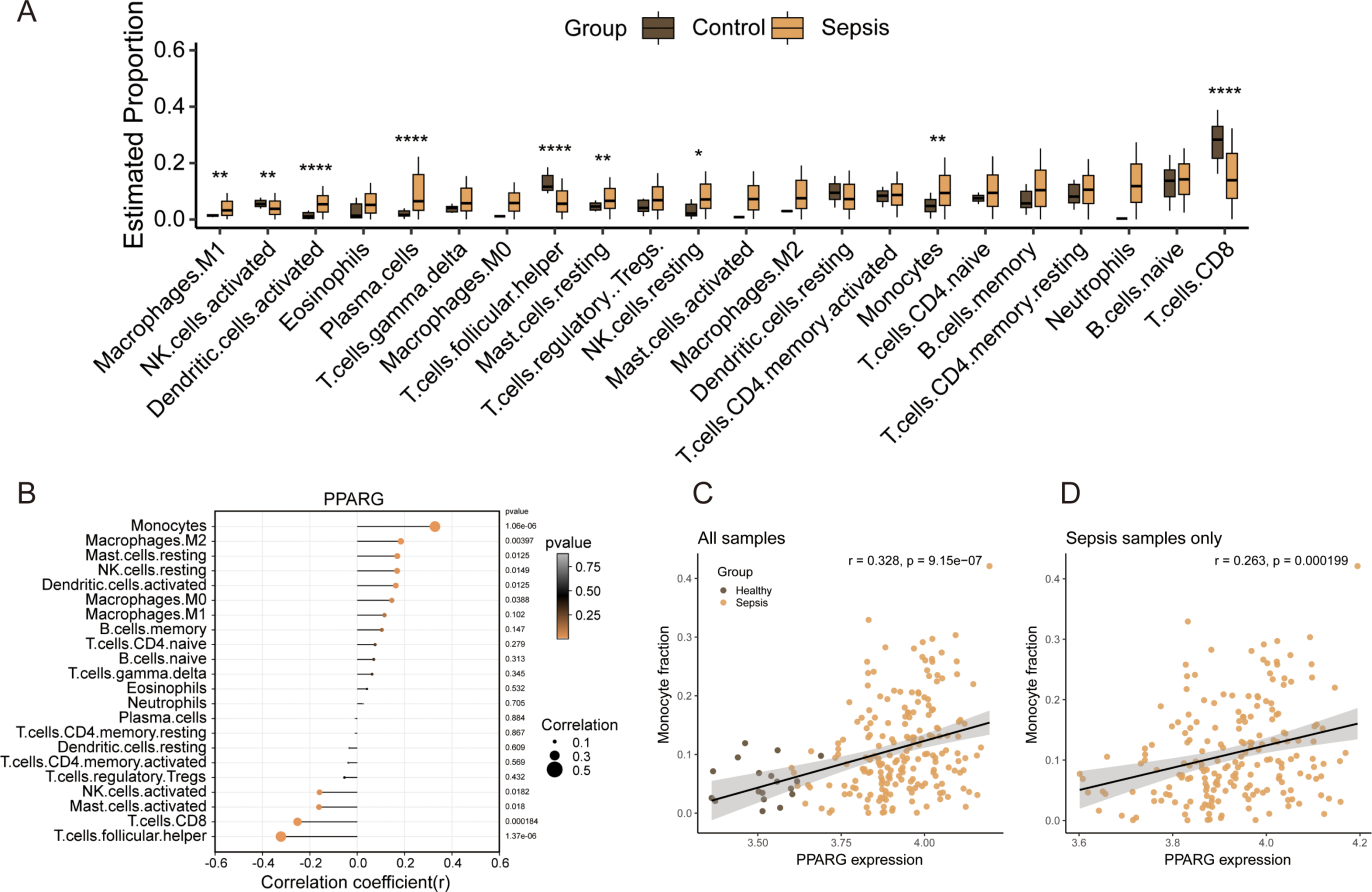
Immune cell composition in sepsis and its association with *PPARG* expression. **(A)** Box plots showing the estimated proportions of 22 immune cell subsets inferred by CIBERSORT in sepsis and healthy control samples. **(B)** Lollipop plot showing Spearman correlations between *PPARG* expression and the estimated proportions of the 22 immune cell subsets. The x-axis indicates the correlation coefficient (r); dot size represents the absolute correlation magnitude, and color indicates statistical significance. **(C)** Scatter plot showing the correlation between *PPARG* expression and monocyte proportion across all samples in GSE236713. **(D)** Scatter plot showing the correlation between *PPARG* expression and monocyte proportion within sepsis samples alone. Group differences in **(A)** were assessed by the Wilcoxon rank-sum test. Correlations in **(B-D)** were assessed by Spearman correlation. ns, *P* ≥ 0.05; *P < 0.05; **P < 0.01; ***P < 0.001.

Given that immune dysregulation is a key feature of sepsis (Girardis et al., 2024), CIBERSORT was used to estimate the relative proportions of 22 immune cell subsets from whole-blood transcriptomic data, and immune cell composition was compared between patients with sepsis and healthy controls (**Figure 6A**). Compared with controls, the sepsis group showed significantly higher proportions of monocytes, M1 macrophages, activated dendritic cells, plasma cells, and resting mast cells, whereas the proportions of natural killer (NK) cells, follicular helper T cells, and CD8+ T cells were significantly lower. The association between *PPARG* expression and immune cell composition was then assessed by correlating *PPARG* expression with the estimated proportions of the 22 immune cell subsets (**Figure 6B**). PPARG was significantly correlated with multiple immune cell subsets, showing the strongest positive correlation with monocyte proportion (*r* = 0.328, *P* = 1.06 × 10^-6^), followed by M2 macrophage proportion (*r* = 0.184, *P* < 0.005). To determine whether the association between *PPARG* expression and monocyte proportion was independent of disease status, a multivariable linear regression model including sepsis status as a covariate was performed. The association remained significant after adjustment (*β* = 0.178, 95% CI 0.094–0.262, *P* = 4.47 × 10^-5^; **Supplementary Table 4**). Positive correlations between *PPARG* expression and monocyte proportion were observed both across all samples and within sepsis samples alone (**Figures 6C, D**).

### 3.6 GSEA reveals signaling pathways associated with PPARG expression stratified by CD14

**Figure 7:**
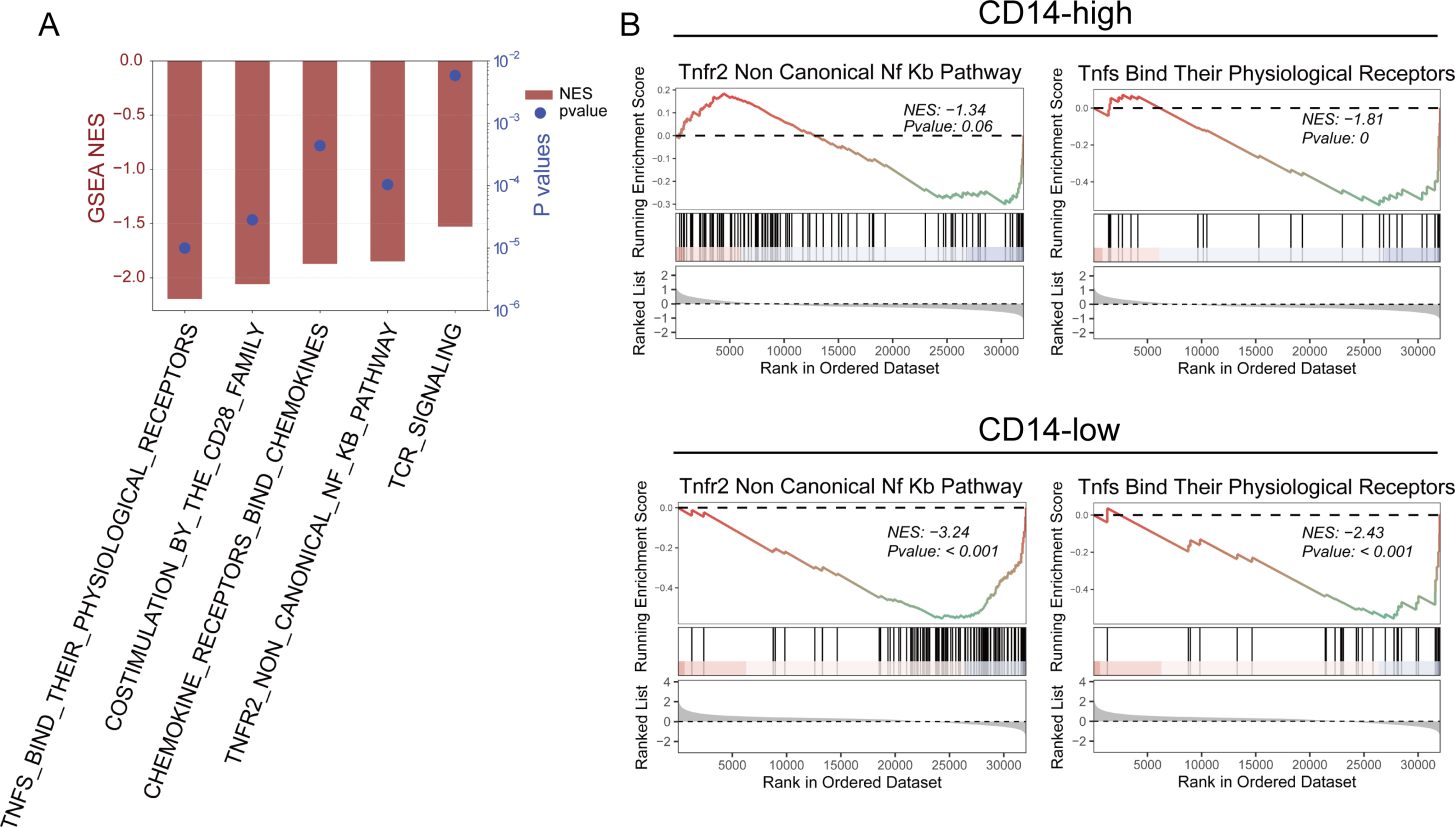
GSEA identifies NF-κB- and TNF/TNFR-related pathways associated with low *PPARG* expression, with enrichment strength differing by *CD14* expression status. **(A)** Bar plot showing normalized enrichment scores (NES) and *P* values for five NF-κB-related Reactome pathways from GSEA comparing *PPARG*-high versus *PPARG*-low samples in GSE236713. Negative NES indicates enrichment in the *PPARG*-low group. **(B)** Representative GSEA enrichment plots for TNFR2_NON_CANONICAL_NF_KB_PATHWAY and TNFS_BIND_THEIR_PHYSIOLOGICAL_RECEPTORS in *PPARG*-high versus *PPARG*-low samples stratified by *CD14* expression (*CD14*-high and *CD14*-low subgroups).

To explore pathways associated with *PPARG* expression, samples in GSE236713 were divided into *PPARG*-high and *PPARG*-low groups using the mean *PPARG* expression as the cutoff. Genes were ranked by log_2_ fold change between the two groups, and GSEA was performed. NF-κB-related gene sets showed significant enrichment in the *PPARG*-low group (**Figure 7A**). Given the key role of CD14-associated inflammatory signaling in sepsis, samples were further stratified into *CD14*-high (*n* = 219) and *CD14*-low (*n* = 135) subgroups, and *PPARG*-based grouping and GSEA were repeated within each stratum. In the *CD14*-low subgroup, TNFR2_NON_CANONICAL_NF_KB_PATHWAY and TNFS_BIND_THEIR_PHYSIOLOGICAL_RECEPTORS showed stronger enrichment in the *PPARG*-low group. In the *CD14*-high subgroup, enrichment of TNFR2_NON_CANONICAL_NF_KB_PATHWAY did not reach statistical significance (*P* = 0.055), whereas the enrichment direction for both pathways remained consistent (**Figure 7B**). Together, these results indicate that enrichment of NF-κB- and TNF/TNFR-related pathways was consistently associated with low *PPARG* expression, although the strength of this association differed across *CD14* expression strata. This difference may reflect variation in the underlying inflammatory context, which could influence the relative strength or statistical detectability of pathway enrichment.

### 3.7 CD14 negatively regulates PPARG expression in LPS-stimulated macrophages

**Figure 8:**
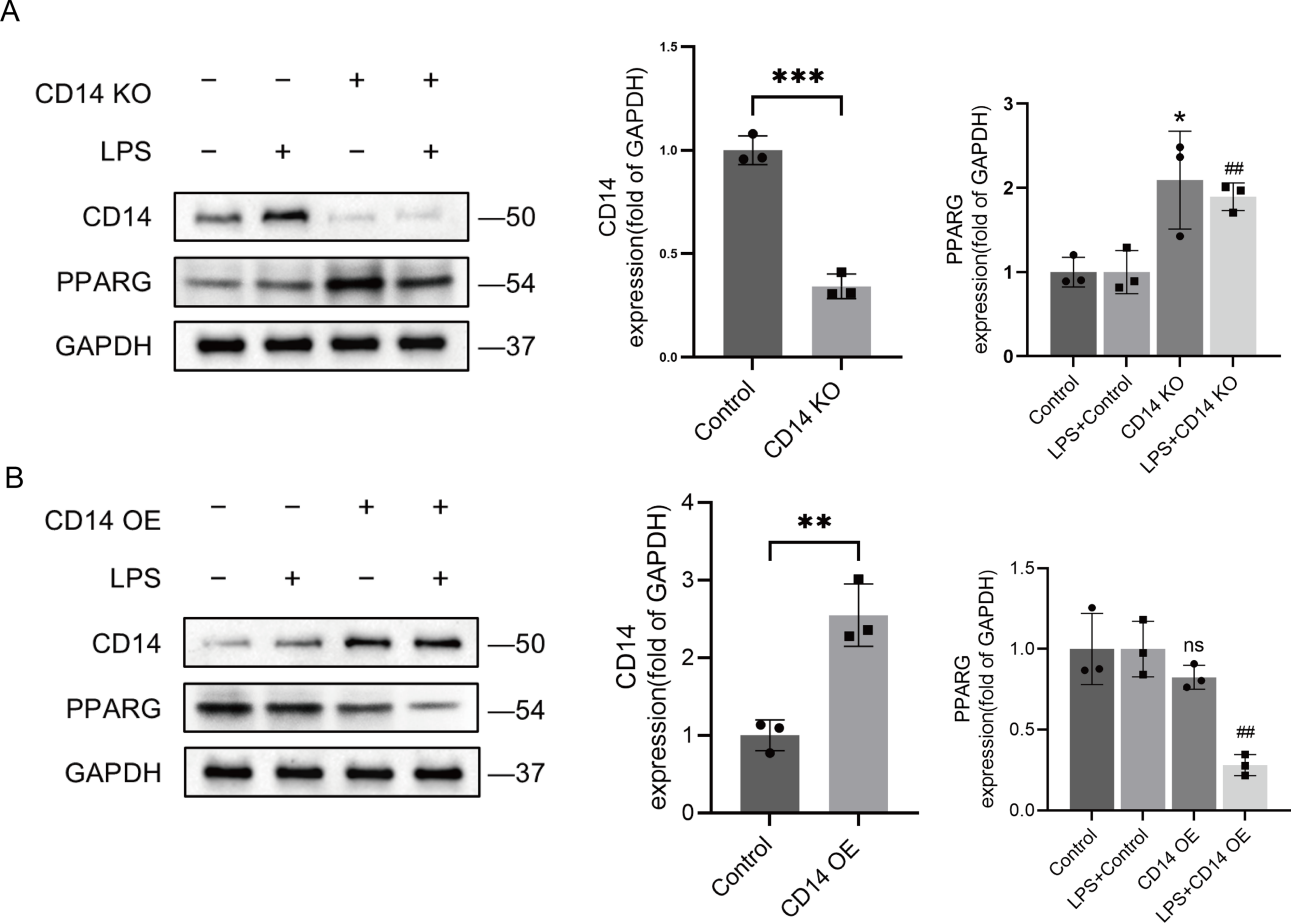
CD14 modulates PPARG expression in LPS-stimulated RAW264.7 macrophages. **(A)** Western blot analysis of CD14 and PPARG protein expression in CD14-KO RAW264.7 cells with or without LPS stimulation (100 ng/mL, 6 h), with GAPDH as a loading control. Bar plots show densitometric quantification of CD14 and PPARG normalized to GAPDH. **(B)** Western blot analysis of CD14 and PPARG protein expression in CD14-OE RAW264.7 cells with or without LPS stimulation (100 ng/mL, 6 h), with GAPDH as a loading control. Bar plots show densitometric quantification of CD14 and PPARG normalized to GAPDH. Statistical comparisons were performed using Student’s t-test. In each panel, * indicates comparisons between CD14-KO **(A)** or CD14-OE **(B)** and the respective NC under basal conditions, and # indicates comparisons between CD14-KO + LPS **(A)** or CD14-OE + LPS **(B)** and NC + LPS. ns, *P* ≥ 0.05; *P < 0.05; **P < 0.01; ***P < 0.001; #*P* < 0.05; ##*P* < 0.01; ###*P* < 0.001.

To examine the effect of CD14 on PPARG expression, CD14-knockout (CD14-KO) and CD14-overexpressing (CD14-OE) RAW264.7 cells were generated. Western blotting confirmed a marked reduction in CD14 protein levels in CD14-KO cells and elevated CD14 protein levels in CD14-OE cells compared with their respective controls (**Figures 8A, B**). PPARG protein levels were assessed by western blotting with densitometric quantification at 6 h after LPS stimulation. In negative control (NC) cells, LPS treatment did not significantly alter PPARG expression (NC vs. NC + LPS). In contrast, PPARG levels were significantly higher in CD14-KO cells than in controls under both basal and LPS-stimulated conditions (**Figure 8A**). Conversely, PPARG expression was reduced in CD14-OE cells, and the CD14-OE + LPS group showed significantly lower PPARG levels than the NC + LPS group (**Figure 8B**). Collectively, these results suggest an inverse relationship between CD14 expression and PPARG levels in LPS-stimulated macrophages.

### 3.8 CD14-dependent regulation of PPARG involves NF-κB signaling

**Figure 9:**
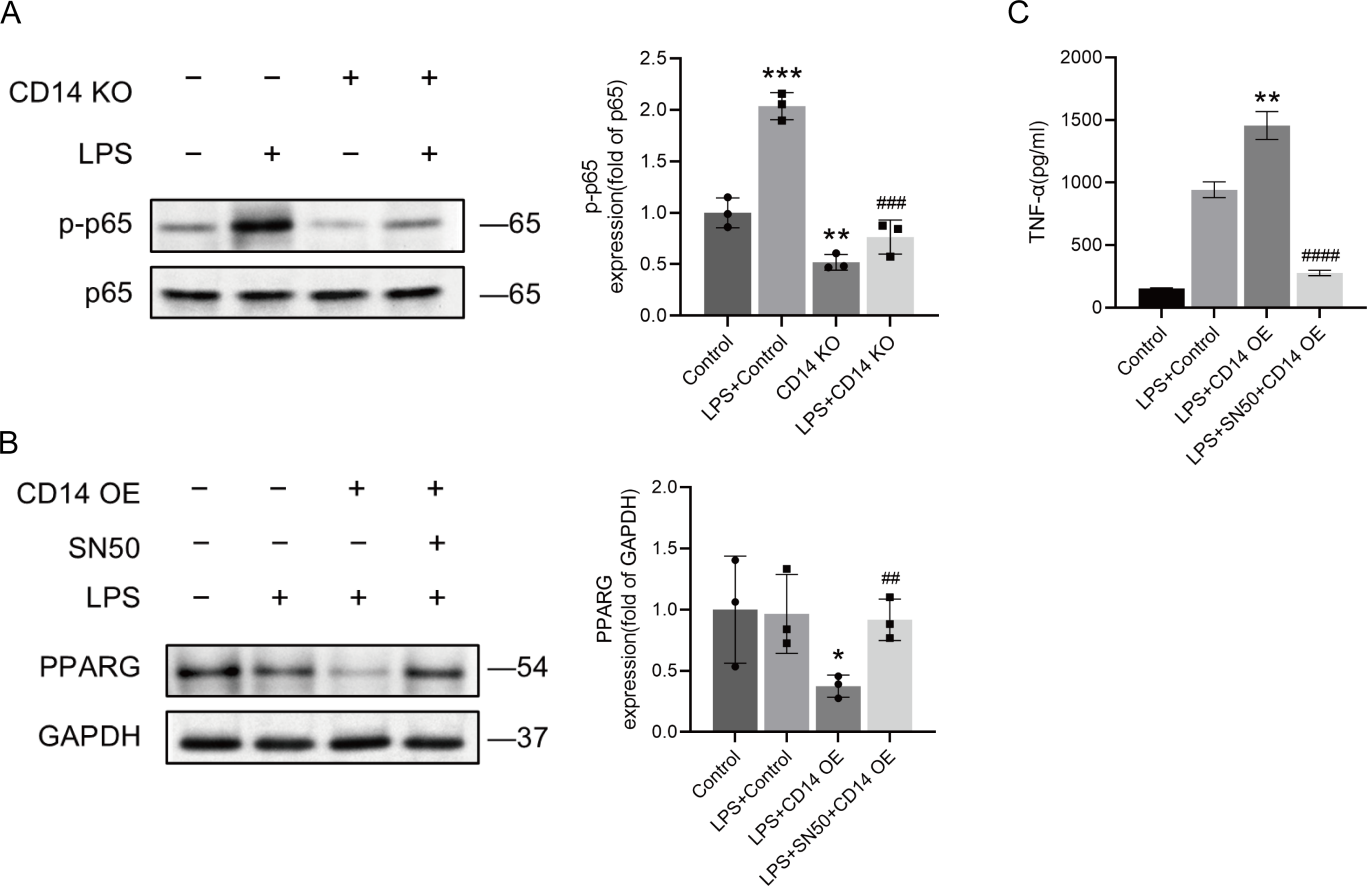
NF-κB signaling mediates CD14-dependent suppression of PPARG in LPS-stimulated RAW264.7 macrophages. **(A)** Western blot analysis of phospho-p65 (p-p65) and total p65 in CD14-KO RAW264.7 cells with or without LPS stimulation (100 ng/mL, 6 h). Bar plot shows densitometric quantification of p-p65 normalized to total p65. **(B)** Western blot analysis of PPARG in CD14-OE RAW264.7 cells treated with LPS (100 ng/mL, 6 h) with or without SN50 pretreatment (10 μM, 4 h). GAPDH was used as a loading control. Bar plot shows densitometric quantification of PPARG normalized to GAPDH. **(C)** TNF-α levels in culture supernatants measured by ELISA under the same conditions as in **(B)**. Statistical comparisons were performed using Student’s t-test. In **(A)**, * indicates comparisons between NC + LPS and NC, and between CD14-KO and NC under basal conditions, and # indicates comparisons between CD14-KO + LPS and NC + LPS. In **(B)** and **(C)**, * indicates comparisons between CD14-OE + LPS and NC + LPS, and # indicates comparisons between CD14-OE + SN50 + LPS and CD14-OE + LPS. ns, *P* ≥ 0.05; *P < 0.05; **P < 0.01; ***P < 0.001; #*P* < 0.05; ##*P* < 0.01; ###*P* < 0.001. Data are presented as mean ± SD from three independent experiments; dots represent individual biological replicates (*n* = 3).

To explore the mechanism by which CD14 is associated with reduced PPARG expression, the involvement of NF-κB signaling was examined. Western blotting showed low basal phosphorylation of p65 (p-p65) in NC cells. After 6 h of LPS stimulation, p-p65 levels increased markedly in NC + LPS cells, whereas total p65 levels remained largely unchanged. In CD14-KO cells, the LPS-induced increase in p-p65 was substantially attenuated and was not significantly different from that in unstimulated cells (**Figure 9A**), indicating that CD14 is required for efficient LPS-induced NF-κB activation in this model.

To determine whether NF-κB mediates the CD14-dependent suppression of PPARG, the NF-κB/Rel nuclear translocation inhibitor SN50 was applied in CD14-OE cells. PPARG protein levels were significantly lower in the CD14-OE + LPS group than in the NC + LPS group. SN50 pretreatment (CD14-OE + SN50 + LPS) significantly increased PPARG levels compared with CD14-OE + LPS (**Figure 9B**), suggesting that inhibition of NF-κB nuclear translocation partially reverses CD14 overexpression-associated PPARG downregulation.

To further verify the inhibitory effect of SN50 on NF-κB pathway output, TNF-α secretion was measured by ELISA. TNF-α levels were significantly elevated in the CD14-OE + LPS group and were significantly reduced following SN50 pretreatment (CD14-OE + SN50 + LPS) (**Figure 9C**), supporting effective inhibition of LPS-induced NF-κB signaling by SN50.

## 4 Discussion

In this study, we developed an integrated workflow linking data-driven biomarker discovery with targeted experimental validation. Using the GSE236713 whole-blood transcriptomic dataset, CD14-associated molecular programs in sepsis were characterized by combining differential expression analysis, WGCNA, and machine learning-based feature selection, together with analyses of clinical severity and short-term outcomes. Our approach focuses on host-response transcriptional biomarkers derived from whole blood. Within this framework, *PPARG* emerged as a prioritized diagnostic biomarker among the five feature genes, and a five-feature-gene ANN classifier (*MMP9*, *PPARG*, *C1QC*, *MS4A4A*, and *ARG1*) demonstrated strong discrimination with external validation in GSE65682. These computational findings were then translated into testable hypotheses *in vitro*. In LPS-stimulated RAW264.7 macrophages, CD14 knockout increased PPARG expression, whereas CD14 overexpression suppressed it. CD14 deficiency also attenuated LPS-induced p65 phosphorylation, and the NF-κB/Rel nuclear translocation inhibitor SN50 partially restored PPARG expression in CD14-overexpressing cells while reducing TNF-α secretion. Collectively, these results support a potentially useful blood-based diagnostic signature and provide supportive evidence that reduced PPARG expression is linked to CD14/NF-κB-associated inflammatory signaling, offering a plausible connection between innate immune activation and immunometabolic dysregulation in sepsis.

### 4.1 Clinical and biological interpretation of PPARG in sepsis

Peroxisome proliferator-activated receptor gamma (PPARG) is a nuclear receptor that integrates immunoregulatory and metabolic programs. Previous studies indicate that PPARG can repress pro-inflammatory gene transcription via transrepression and counteract inflammatory transcriptional circuits, including NF-κB signaling (Bougarne et al., 2018; Pokimica and García-Conesa, 2018). In macrophages, PPARG activation has also been linked to polarization toward an M2-like phenotype (Christofides et al., 2021). Beyond immunity, PPARG regulates fatty acid uptake, lipid biosynthesis, and lipoprotein metabolism (Li et al., 2024), and may therefore reflect systemic alterations in immunometabolic coupling during sepsis. These established functions make PPARG a biologically plausible blood-based diagnostic biomarker. Consistent with this premise, PPARG showed strong single-gene diagnostic performance, and the five-feature-gene ANN classifier maintained strong performance in the independent GSE65682 cohort. PPARG was also associated with SOFA score and ICU short-term outcome risk in the discovery cohort, while its association with 28-day survival in GSE65682 showed only a non-significant trend. These findings support the diagnostic relevance of PPARG, whereas its relationships with disease severity and prognosis should be interpreted cautiously and validated in independent cohorts. Time-stratified analysis further suggested that PPARG remained dysregulated during the early ICU course, although modest short-term temporal variation may exist.

An important interpretive issue is that higher PPARG expression was associated with greater disease severity and increased short-term outcome risk in the discovery cohort, despite the recognized anti-inflammatory functions of PPARG and the reported protective effects of PPARG agonists in preclinical sepsis models (Koutroulis et al., 2019). Rather than indicating a direct pathogenic effect of PPARG itself, this apparently paradoxical pattern may reflect at least two non-mutually exclusive mechanisms. First, increased PPARG expression may represent a reactive or compensatory immunoregulatory response to severe systemic inflammation and immunometabolic stress, rather than an indicator of effective anti-inflammatory control. In severe sepsis, immune suppression and metabolic reprogramming can coexist with ongoing inflammatory injury, and the upregulation of immunoregulatory programs has been linked to greater severity and worse outcomes (Liu et al., 2022). Second, because our transcriptomic analyses were based on bulk whole-blood data, the observed increase in PPARG expression may partly reflect shifts in circulating immune cell composition rather than cell-intrinsic transcriptional activation alone. This interpretation is supported by our finding that PPARG was most strongly associated with monocyte proportion, and by prior work showing that changes in myeloid cell abundance can substantially influence bulk whole-blood gene expression signals (O’Connell et al., 2019). In addition, increased PPARG mRNA abundance does not necessarily translate into increased PPARG pathway activity (Kd et al., 2018; Potts et al., 2025). Under intense inflammatory stress, PPARG function may become dissociated from transcript abundance through mechanisms extending beyond transcriptional regulation, including altered splicing and post-translational modification (Pascual et al., 2005; Cataldi et al., 2021). Thus, elevated PPARG expression in severe sepsis may coexist with insufficient downstream anti-inflammatory activity. Given the established role of PPARG in monocyte/macrophage immunometabolic regulation (Peng et al., 2025), our findings are more consistent with PPARG acting as a blood-based biomarker reflecting disease state, myeloid cell context, and systemic immunometabolic stress than with a simple linear protective readout.

### 4.2 Potential involvement of the CD14/NF-κB/PPARG axis

Beyond its clinical associations, our data provide mechanistic clues linking PPARG to upstream innate immune signaling, particularly an inverse relationship between CD14 and PPARG expression. CD14 is classically recognized as a co-receptor for TLR4 that facilitates LPS sensing and triggers downstream pro-inflammatory signaling (Chen et al., 2020). However, how CD14-driven signaling interfaces with counter-regulatory anti-inflammatory programs remains less clearly defined. In LPS-stimulated RAW264.7 macrophages, CD14 knockout was associated with higher PPARG expression under both basal and stimulated conditions, whereas CD14 overexpression further reduced PPARG protein abundance following LPS challenge. Together, these data suggest that CD14 levels can negatively modulate PPARG expression in macrophages, particularly under LPS-stimulated inflammatory conditions, and raise the possibility that heightened CD14 signaling may coincide with repression of metabolic and anti-inflammatory regulatory programs.

To further interpret this regulatory relationship, we combined pathway-level and cell-context analyses with *in vitro* perturbation experiments. GSEA indicated that NF-κB- and TNF/TNFR-related gene sets were enriched in samples with low *PPARG* expression. Consistent with these observations, CD14 deficiency attenuated LPS-induced p65 phosphorylation *in vitro*, and pharmacologic inhibition of NF-κB nuclear translocation with SN50 in CD14-overexpressing cells partially restored PPARG expression. Together, these findings support the involvement of NF-κB signaling in CD14-associated suppression of PPARG. CIBERSORT-based analyses indicated that *PPARG* expression was most strongly correlated with the inferred proportion of monocytes, suggesting that myeloid cells may represent an important cellular context for this regulatory relationship. This finding also supports the relevance of a macrophage-based model for mechanistic validation. Collectively, these data are consistent with a model in which CD14/NF-κB-associated inflammatory signaling contributes to PPARG suppression under sepsis-related inflammatory conditions.

### 4.3 Limitations

Despite these findings, several limitations should be considered. First, although the diagnostic performance of our ANN model was externally validated in GSE65682, the model was developed and validated primarily for distinguishing sepsis from healthy controls rather than from clinically relevant inflammatory comparator groups. In real-world practice, differentiating sepsis from non-infectious systemic inflammatory response syndrome (SIRS), trauma, postoperative inflammatory states, or other forms of non-septic critical illness represents a more important diagnostic challenge. The absence of validation in such comparator populations therefore limits the immediate clinical applicability and diagnostic specificity of the current five-gene panel. The present study also did not include direct performance comparison with established sepsis biomarkers such as CRP or PCT, because such measurements were not available in the public transcriptomic datasets used here. Although clinical relevance was supplemented through external validation and SOFA- and outcome-related analyses, these findings do not substitute for comparative evaluation against conventional biomarkers. Accordingly, the current findings should be interpreted as evidence of discriminative potential within the present study framework, rather than as establishing PPARG or the five-gene panel as a clinically validated standard biomarker approach. In addition, all participants in the discovery and external validation datasets were of European ancestry. Therefore, the generalizability of PPARG and the five-gene panel to populations with different ancestral backgrounds remains uncertain, and validation in ethnically diverse cohorts is required before broader clinical application.

Second, mechanistic validation was performed primarily in an LPS-stimulated RAW264.7 macrophage model. As RAW264.7 is a murine macrophage cell line, this system does not fully recapitulate the species-specific transcriptional context or the cellular complexity of human whole blood, nor can it capture the pathological context of *in vivo* multi-organ interactions in sepsis. These experiments should therefore be interpreted as mechanistic support for the potential involvement of the CD14/NF-κB/PPARG axis, rather than as direct validation in human blood cells.

Third, our transcriptomic analyses were based on bulk whole-blood data. Although the discovery cohort included D1/D2/D5 sampling and supplementary analyses suggested that CD14 expression was relatively stable across these early time points, *PPARG* showed a modest difference between D1 and D5. Short-term temporal heterogeneity, together with shifts in immune cell composition, may therefore have influenced the observed expression patterns and their clinical interpretation. Furthermore, because the PPI analysis relied on the curated STRING database with a high-confidence interaction threshold, the hub gene prioritization step may have preferentially retained genes with richer prior interaction knowledge, including well-studied immune-related genes. These considerations should be taken into account when interpreting the biological specificity of the identified gene set and the inferred role of PPARG in sepsis. Despite these limitations, this study provides a coherent evidence chain linking transcriptomic analyses with *in vitro* validation and suggests that CD14/NF-κB-associated regulation of PPARG may be involved in sepsis-related immunometabolic dysregulation. This evidence supports the continued investigation of PPARG as a biomarker candidate in sepsis.

### 4.4 Future directions

Future studies should further evaluate the translational applicability of PPARG and the five-gene panel as whole-blood transcript-based tools for host-response assessment in sepsis, with potential relevance for severity evaluation. Realizing this potential would require validation at multiple levels. Analytically, this would include assay standardization across platforms and laboratories, reference gene selection, and establishment of clinically meaningful expression thresholds. Clinically, it would require prospective evaluation in well-characterized multicenter sepsis cohorts, assessment in relevant comparator populations such as non-infectious SIRS, trauma, postoperative inflammatory states, and other acute infections, and demonstration of incremental value beyond established clinical scores and biomarkers. Additional confirmation in independent, prospectively collected cohorts will be important. Ideally, such cohorts should include multiple sites, geographic regions, ethnically diverse populations, and matched conventional biomarker measurements to further assess generalizability, diagnostic specificity, relative diagnostic performance, and potential clinical utility.

In parallel, future studies should strengthen the biological validation of the CD14/NF-κB/PPARG axis using human primary monocytes/macrophages, whole-blood-based systems, and *in vivo* sepsis models. Genetic or pharmacological perturbation strategies may help to more rigorously confirm the CD14/NF-κB/PPARG regulatory relationship and to clarify its impact on inflammatory responses, organ injury, and clinical outcomes.

Cell type-resolved and longitudinal investigations will also be important for clarifying the context-dependent functions of PPARG in sepsis. Approaches such as single-cell transcriptomics or cell sorting, together with additional temporal validation and longitudinal sampling, may help define the expression patterns and functional states of PPARG across immune cell subsets and across different stages of sepsis progression. Together, these future efforts will be important for defining the biological relevance, diagnostic specificity, and potential clinical utility of PPARG and the five-gene panel in sepsis more rigorously.

## Data Availability

Publicly available datasets were analyzed in this study. This data can be found here: https://www.ncbi.nlm.nih.gov/geo/query/acc.cgi?acc=GSE236713 https://www.ncbi.nlm.nih.gov/geo/query/acc.cgi?acc=GSE65682.
