# Supplementary material for: Machine learning identifies PPARG as a diagnostic biomarker for sepsis linked to CD14/NF-κB signaling: integrated transcriptomics and experimental validation
Source: Front Cell Infect Microbiol. 2026 May 28;16:1800050. doi: 10.3389/fcimb.2026.1800050 (PMC13253277; doi:10.3389/fcimb.2026.1800050)
Supplement: Supplementary file 1 [file DataSheet1.zip › Supplementary/Supplementary tables/Supplementary Table S3.docx]

| **Supplementary Table S3. Baseline Clinical Characteristics of Study Cohorts** | | | | |
| --- | --- | --- | --- | --- |
|  |  |  |  |  |
|  | **Discovery Cohort (GSE236713)** | | **Validation Cohort (GSE65682)** | |
| **Variable** | **Sepsis (n = 143)** | **Healthy Controls (n = 30)** | **Sepsis (n = 760)** | **Healthy Controls (n = 42)** |
| ***Demographics*** | | | | |
| Age, median (IQR), years | 68 (59–76) | 42 (33–48) | 63 (53–72) | 37 (30–63) |
| Male sex, n (%) | 72 (50.7%) | 9 (30.0%) | 446 (58.7%) | 24 (57.1%) |
| Ethnicity | European | European | European | European |
| ***Source of Infection, n (%)*** | | | | |
| Pulmonary | 84 (58.7%) | — | 192 (25.3%) † | — |
| Abdominal | 59 (41.3%) | — | 51 (6.7%) | — |
| Other / Not recorded | — | — | 517 (68.0%) | — |
| ***Disease Severity*** | | | | |
| SOFA score, median (IQR) | 16 (13–18) ‡ | — | Not available | — |
| ***Comorbidity*** | | | | |
| Diabetes mellitus, n (%) | Not available | — | 94/410 (22.9%) § | — |
| ***Clinical Outcome*** | | | | |
| ICU / 28-day mortality, n (%) | 29/126 (23.0%) ¶ | — | 114/479 (23.8%) # | — |
|  |  |  |  |  |
|  |  |  |  |  |
| *† Pulmonary sepsis in GSE65682 includes community-acquired pneumonia (CAP, n=108) and hospital-acquired pneumonia (HAP, n=84).* | | | | |
| *‡ SOFA scores in GSE236713 were recorded across all sampling time points (D1/D2/D5); 20 of 324 samples had missing SOFA values.* | | | | |
| *§ Diabetes mellitus data available for 410 of 760 sepsis patients in GSE65682.* | | | | |
| *¶ ICU mortality in GSE236713: outcome data available for 126 of 143 sepsis patients.* | | | | |
| *# 28-day mortality in GSE65682: outcome data available for 479 of 760 patients.* | | | | |
| *—, not applicable. IQR, interquartile range. SOFA, Sequential Organ Failure Assessment.* | | | | |
