# Supplementary material for: Machine learning identifies PPARG as a diagnostic biomarker for sepsis linked to CD14/NF-κB signaling: integrated transcriptomics and experimental validation
Source: Front Cell Infect Microbiol. 2026 May 28;16:1800050. doi: 10.3389/fcimb.2026.1800050 (PMC13253277; doi:10.3389/fcimb.2026.1800050)
Supplement: Supplementary file 1 [file DataSheet1.zip › Supplementary/Supplementary tables/Supplementary Table S4.docx]

**Supplementary Table S4**. Multivariable linear regression of monocyte proportion on PPARG expression and sepsis status.

| Variable | β | 95% CI | p value |
| --- | --- | --- | --- |
| PPARG expression | 0.178 | [0.094,0.262] | 4.47 x 10 ^-5^ |
| Sepsis status | -0.016 | [-0.065,0.034] | 0.540 (23.8%) |
