# Supplementary figures and images for: Machine learning identifies PPARG as a diagnostic biomarker for sepsis linked to CD14/NF-κB signaling: integrated transcriptomics and experimental validation
Source: Front Cell Infect Microbiol. 2026 May 28;16:1800050. doi: 10.3389/fcimb.2026.1800050 (PMC13253277; doi:10.3389/fcimb.2026.1800050)

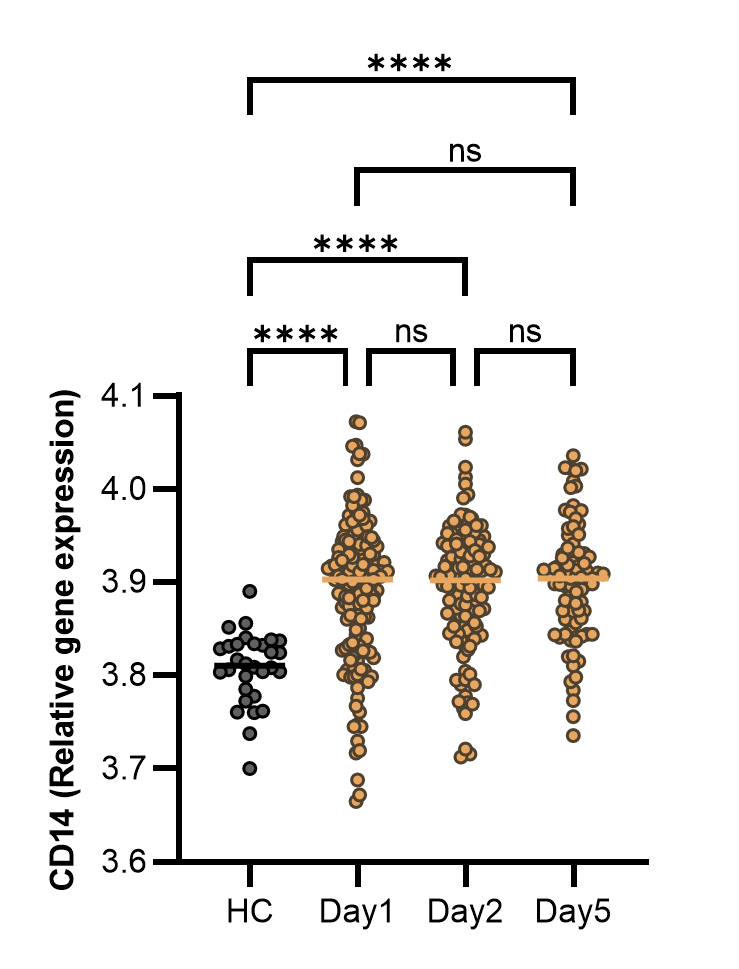

Supplement: Supplementary file 1 [file DataSheet1.zip › Supplementary/Supplementary figures/Supplementary Figure S1.tif]

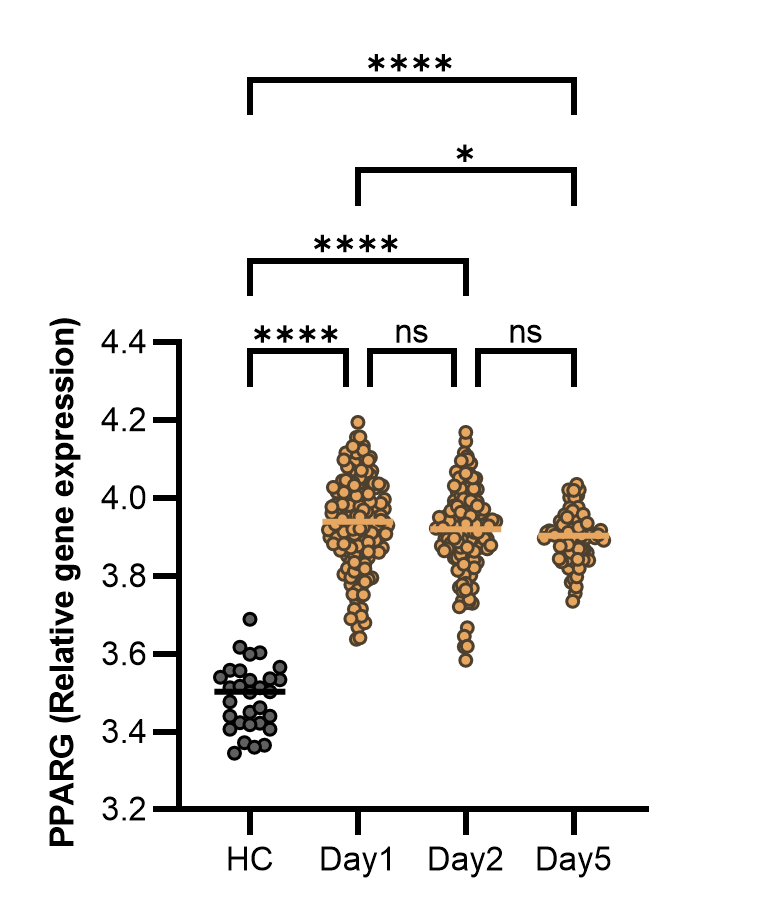

Supplement: Supplementary file 1 [file DataSheet1.zip › Supplementary/Supplementary figures/Supplementary Figure S2.tif]

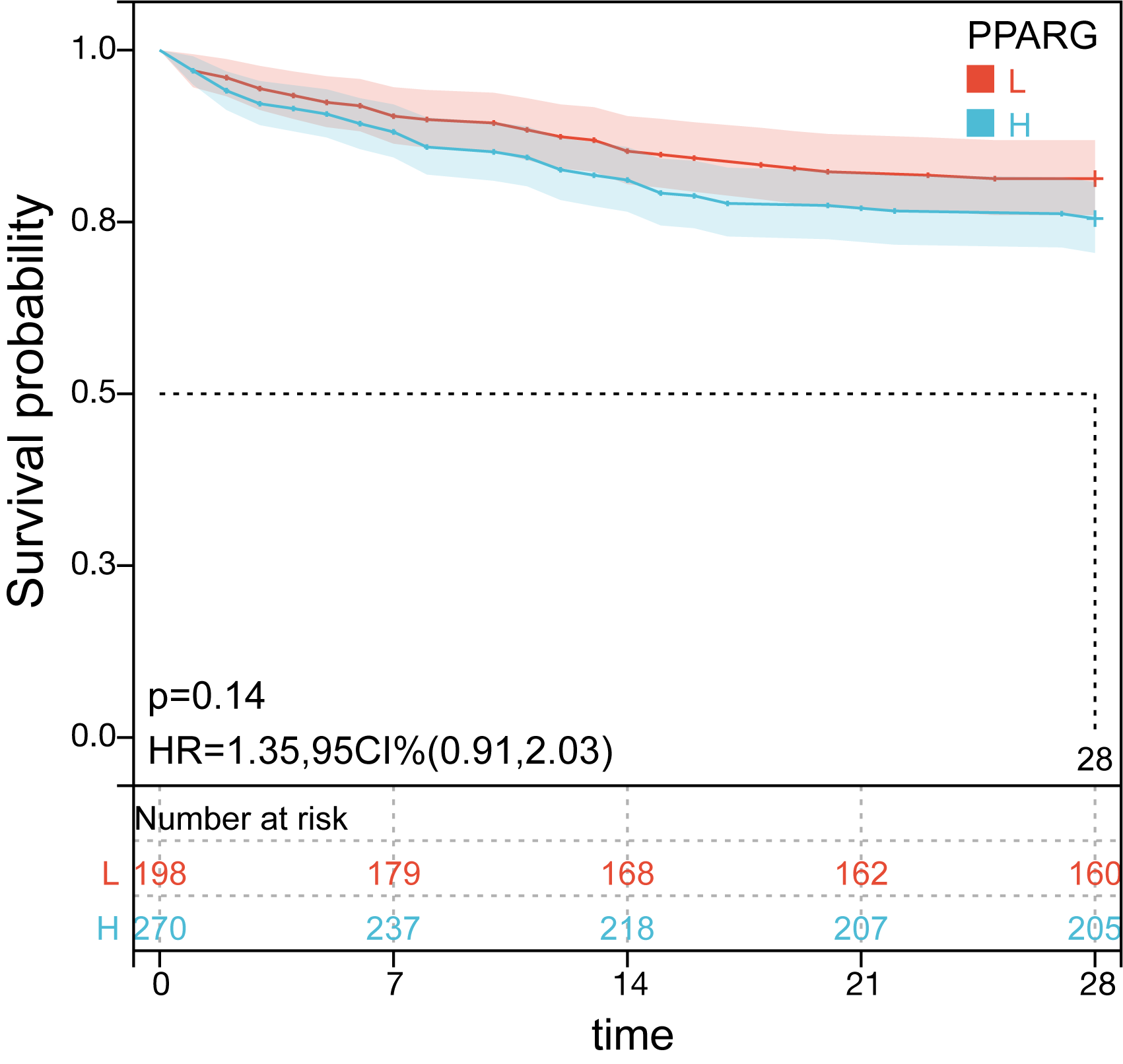

Supplement: Supplementary file 1 [file DataSheet1.zip › Supplementary/Supplementary figures/Supplementary Figure S3.tif]
